# Ferroptosis: A New Promising Target for Lung Cancer Therapy

**DOI:** 10.1155/2021/8457521

**Published:** 2021-09-25

**Authors:** Rui Xiong, Ruyuan He, Bohao Liu, Wenyang Jiang, Bo Wang, Ning Li, Qing Geng

**Affiliations:** Department of Thoracic Surgery, Renmin Hospital of Wuhan University, Wuhan, China

## Abstract

Ferroptosis is a new type of regulatory cell death that differs from autophagy, apoptosis, necrosis, and pyroptosis; it is caused primarily by the accumulation of iron and lipid peroxides in the cell. Studies have shown that many classical signaling pathways and biological processes are involved in the process of ferroptosis. In recent years, investigations have revealed that ferroptosis plays a crucial role in the progression of tumors, especially lung cancer. In particular, inducing ferroptosis in cells can inhibit the growth of tumor cells, thereby reversing tumorigenesis. In this review, we summarize the characteristics of ferroptosis from its underlying basis and role in lung cancer and provide possible applications for it in lung cancer therapies.

## 1. Introduction

Lung cancer is among the leading causes of death worldwide and is the primary reason people die in China; it resulted in more than 2.8 million Chinese deaths in 2015, with over 40% of the total number related to lung adenocarcinomas [1]. Gererally, lung cancer can be divided into small-cell lung cancer and non-small-cell lung cancer (NSCLC). The latter includes adenocarcinoma (ADC) and squamous cell carcinoma (SCC), accounting for 80% to 85% of all lung cancer cases [2]. The serum, bronchoalveolar lavage fluid, and exhaled air condensate of patients with lung cancer usually contain elevated ferritin levels [3–5]. Transferrin receptor protein 1 (TFR1) is highly expressed in 88% of NSCLCs [6], which suggests that lung cancer cells could increase iron intake by enhancing the effects of the transferrin protein (TF) and TFR.

Ferroptosis is a new type of regulatory cell death that is caused by iron-dependent lipid peroxidation. This new form of regulatory cell death is characterized primarily by reduced cell volume and increased mitochondrial membrane density, without the typical manifestations of apoptosis and necrosis [7]. We are yet to find a study that reports ferroptosis in human lung cancer. However, based on the characteristics of ferroptosis, we can boldly guess that an increase in iron intake by lung cancer cells is likely to cause ferroptosis, with ferroptosis potentially one of the many forms of death for lung cancer cells. So, combining ferroptosis with chemotherapy, radiation, or immunotherapy to treat lung cancer could improve anticancer effects.

The key challenge in cancer therapy is how to effectively kill cancer cells without affecting healthy cells. The main reason for cancer therapy resistance is that the cancer cells often have defects in the cell death actuator mechanism. Cancer cells require significant amounts of iron to survive and grow compared to normal, noncancer cells. This dependence on iron makes cancer cells more susceptible to iron-catalyzed cell death, also known as ferroptosis [8]. Therefore, targeting ferroptosis could provide a new and promising approach to killing therapy-resistant cancers.

## 2. The Mechanism of Ferroptosis

### 2.1. Iron Overload

Iron is a basic trace element in the body, which is required for normal cells to perform various biological functions. For example, iron is essential for DNA biosynthesis, cell-cycle progression, and protein function and is needed for cell proliferation [9]. Cells absorb circulating TF-Fe^3+^ through transferrin receptor 1 (TFR1), releasing Fe^3+^ in the endosome where it is reduced to Fe^2+^, transferred into the cytoplasm, and stored in the labile iron pool. Excess Fe^2+^ in cells can be stored in ferritin or excreted from the cell via ferroportin, in which Fe^2+^ is oxidized into Fe^3+^ and binds to TF in the blood [10]. These processes ensure iron metabolism in a healthy human body remains balanced.

Iron metabolism dysfunction is closely linked to ferroptosis. Ferroptosis is inhibited when intracellular iron is depleted by the iron chelating agent deferroamine (DFO), or reactive oxygen species (ROS) is inhibited by ferstatin 1 (Fer-1). Conversely, ferroptosis sensitivity was significantly increased when intracellular iron levels were increased by ferric ammonium citrate, iron chloride hexahydrate, or iron-bound TF [7, 11]. In addition, a decrease in ferritin can stimulate the release of Fe^2+^ in large quantities, and iron overload can enhance the production of intracellular ROS via the Fenton reaction. Excessive ROS triggers lipid peroxidation within the cell membrane, with disproportionate lipid peroxide deposition promoting ferroptosis [12, 13]. Therefore, the intracellular iron content has an important role in maintaining cell homeostasis.

### 2.2. Lipid Peroxidation

Lipid peroxidation is the ultimate executor of ferroptosis during iron overload. Glutathione peroxidase 4 (GPX4) is a core enzyme that regulates lipid peroxidation and acts as a phospholipid hydroperoxidase to reduce lipid peroxides to lipid alcohols [14, 15]. Therefore, GPX4 plays a critical role in maintaining lipid homeostasis in the cell and preventing the accumulation of lipid ROS, ultimately blocking the kind of an oxidative, iron-dependent, nonapoptotic mode of programmed cell death termed ferroptosis [7, 16].

Glutathione (GSH), a necessary cofactor for the antioxidant response of GPX4, can cycle between reduced (GSH) and oxidized (GSSG) states, enabling this metabolite to participate in redox biochemical reactions [17, 18]. Therefore, the targeted regulation of GSH is also an important mechanism of ferroptosis.

System x_c_^−^ is a cell-surface cysteine-glutamate antiporter, which is composed of the 12-pass transmembrane transporter protein SLC7A11 (xCT) and linked to the single-pass transmembrane regulatory subunit SLC3A2 via a disulfide bridge [19]. This antiporter can mediate extracellular cysteine input and intracellular glutamate output, thereby promoting the synthesis of GSH in cells and protecting cells from oxidative stress [20]. Hence, inhibiting system x_c_^−^ can reduce the production of GSH, indirectly weaken the effect of GPX4, and increase the deposition of lipid peroxides, leading to ferroptosis.

### 2.3. Selective Ferroptosis Inducers

At present, there are two types of classical selective ferroptosis inducers: type I and type II inducers. Type I ferroptosis inducers refer to the inhibitors of system x_c_^−^, such as erastin, sulfasalazine, and sorafenib, that can reduce the acquisition of cystine by cells, hindering the synthesis of glutathione, a substrate of GPX4, and triggering the accumulation of lipid reactive oxygen species and subsequently, ferroptosis. Type II ferroptosis inducers denote GPX4 inhibitors, such as RSL3 and FIN56, that can inhibit the synthesis of GPX4 directly and eventually cause ferroptosis [21].

## 3. Ferroptosis in Lung Cancer-Associated Signaling Pathways

### 3.1. Lymphoid-Specific Helicase (LSH)

Lymphoid-specific helicase (LSH) is vital for the normal development of mammals because it establishes the normal levels and patterns of DNA methylation, and it belongs to the SNF2 family of chromatin remodeling ATPases [22–25]. Moreover, LSH has the function of maintaining genomic stability of mammalian somatic cells [26, 27]. Jiang et al. [28] demonstrated a molecular basis for the Egl Nine Homolog 1 (EGLN1)/c-Myc-mediated induction of the LSH expression that inhibits ferroptosis, and this discovery can be applied to develop therapeutic strategies targeting ferroptosis in the cancer therapy. The authors showed that EGLN1 and c-Myc could directly activate the expression of LSH via inhibiting HIF-1*α*, with LSH functioning as an oncogene and regulating ferroptosis in A549 and H522 cell lines. Mechanistically, LSH decreased the intracellular levels of iron and lipid ROS, ultimately inhibited ferroptosis. Also, to explore the role of LSH in lung cancer in vivo, the authors assessed tumor formation in nude mice utilizing a xenograft model and found that injecting the mice with H358-LSH cells caused a significant increase in tumor size, tumor volume, and tumor weight. Therefore, LSH has an inhibitory effect on ferroptosis and is upregulated by the EGLN1/c-Myc axis. Similarly, Yang et al. [29] established that LSH interacts with and stabilizes the GINS4 transcript that promotes tumorigenesis in NSCLC; LSH induces the GINS4 expression and stabilizes it. The Go, Ichi, Nii, and San complex subunit 4 (GINS4), a member of the GINS family of proteins, is essential for the initiation of DNA replication [30, 31]. GINS4 has been proved to be involved in early embryogenesis in mice and and has the properties of maintaining cell cycle progression and genome integrity [31, 32], suggesting that it has a role in tumorigenesis. In the Yang et al. study [29], the authors also found that the overexpression of GINS4 promoted cancer cell growth, migration, and invasion in lung cancer cell lines PC9 and H358. This overexpression was equally proof that GINS4 had an important role in lung cancer progression in the xenograft nude mouse model. Moreover, GINS4 knockdown inhibited cancer progression in H1299 cells, serving as evidence that knocking down GINS4 inhibits cancer progression in vivo. So far, no study has demonstrated that ferroptosis in lung cancer cell death is caused by the inhibition of GINS4. But based on the fact that LSH can inhibit ferroptosis in lung cancer cells and LSH is an upstream signaling molecule of GINS4, it can be speculated that the LSH/GNIS4 axis plays a potentially negative regulatory role in lung cancer cell ferroptosis.

### 3.2. NFS1

NFS1 is an essential enzyme in eukaryotes which can harvest sulfur from cysteine for the biosynthesis of iron-sulfur clusters (ISCs). The latter are protein cofactors capable of sensing oxidative damage in various human enzymes [33–35]. Alvarez et al. [36] found that NFS1 undergoes positive selection in lung tumors and protects cells from ferroptosis. They examined the NFS1 expression in different types of lung cancer by immunohistochemistry and found that the NFS1 expression was the highest in lung adenocarcinoma, followed by lung squamous cell carcinoma and small cell lung cancer. In addition, they further found that the inhibition of NFS1 was correlated to the inhibition of cysteine transport, which triggered ferroptosis in A549, NCI-H838, and NCI-H460 cell lines, while the induction of iron-starvation response induced by NFS1 promoted ferroptosis. Therefore, NFS1 can regulate ferroptosis in lung cancer cells negatively.

### 3.3. Long Noncoding RNA (LncRNA)

According to Mao et al. [37], lncRNA P53RRA promotes ferroptosis in lung cancer through the nuclear sequestration of p53. The authors observed that P53RRA caused an increase in erastin-induced growth inhibition in H522, SPCA1, and A549 cells, whereas the loss of P53RRA led to a decrease in erastin-induced ferroptosis. Also, P53RRA prompted an upsurge in the intracellular concentrations of iron and lipid ROS in H522, SPCA1, and A549. Additionally, the authors provided evidence that P53RRA plays a tumor suppressor role by activating the p53 pathway in lung cancer cells. It is worth mentioning that Yu et al. [38] used RNA sequencing to uncover key lncRNAs and underlying molecular mechanisms of XAV939-mediated NSCLC growth inhibition. The downregulation of lncRNA MIR503HG induced by XAV939 may serve an important role in NSCLC growth inhibition through the sponging of miR-1273c and regulation of the SOX4 expression. In addition, SLC7A11 downregulation induced by XAV939 may inhibit the development of NSCLC through promoting ferroptosis. LncRNA MIR503HG, therefore, inhibits ferroptosis in lung cancer cells. What is more, Wang et al. [39] established that lncRNA LINC00336 inhibits ferroptosis in A549/H358/PC9/SPC-A-1 lung cancer cell lines by functioning as a competing endogenous RNA.

### 3.4. Nuclear Factor Erythroid 2-Related Factor 2/Heme Oxygenase-1 (Nrf2/HO-1)

Many evidences have shown that the nuclear factor erythroid 2-related factor 2 (Nrf2) overexpression is associated to increased resistance to anticancer therapies and poor survival prognosis [40–42]. Heme oxygenase-1 (HO-1) is one of the target genes of Nrf2 and reduces oxidative stress by reducing the overall ROS production and degrading prooxidants [43]. Gloria et al. [44] found that the combination of the lysosomal instability drug, siramesine, and the dual tyrosine kinase inhibitor, lapatinib, triggered synergistic ferroptosis by reducing HO-1 levels in the A549 lung adenocarcinoma cell line. According to Gai et al. [18], acetaminophen- (APAP-) sensitizing erastin induced ferroptosis by regulating the Nrf2/HO-1 signaling pathway in A549 and H1299 NSCLC cell lines. In vivo, the authors treated subcutaneous xenograft tumors in nude mice with erastin and/or APAP and found that the weight of xenograft tumors in the cotreatment group was much smaller than that in either the APAP or the erastin separate groups. In summary, Nrf2/HO-1 promotes ferroptosis in lung cancer cells.

### 3.5. Serine Threonine Tyrosine Kinase 1/Novel Oncogene with Kinase Domain (STYK1/NOK)

Serine threonine tyrosine kinase 1 (STYK1) and novel oncogene with kinase domain (NOK) are members of the receptor protein tyrosine kinases [45]. 75.4% of NSCLC tissues expressed STYK1, which is related to the pTNM stage of tumor differentiation [46]. An investigation by Lai et al. [47] found that STYK1/NOK correlated with ferroptosis in non-small-cell lung cancer. In terms of mechanism, the overexpression of STYK1 in SW900 lung cancer cell line leads to upregulation of the GPX4 expression, promotes cell proliferation, and alleviates various mitochondrial abnormalities characteristic of ferroptosis, while GPX4 knocdown performed an opposite result. Therefore, we can conclude that STYK1/NOK regulates ferroptosis in lung cancer cells negatively.

### 3.6. Ferroptosis Suppressor Protein 1 (FSP1)

Ferroptosis suppressor protein 1 (FSP1) is a potent ferroptosis-resistance factor, a newly discovered by Bersuker et al. [48]. They found that knocking out FSP1 in the highly resistant H460 cell line resulted in a significant, approximately 100-fold sensitization of the cells to RSL3, while the overexpression of FSP1 in sensitive H1703 and H446 cells resulted in approximately 10-20-fold increase in resistance to RSL3. Furthermore, to determine whether inhibition of FSP1 might be clinically relevant to in vivo tumor sensitization to ferroptosis-activated chemotherapy, Bersuker et al. used ferroptosis-resistant H460 lung cancer cells in a preclinical xenograft tumor mouse model generated with GPX4^KO^ and GPX4^KO^ FSP1^KO^ H460 cell lines [48]. Considering ferrostatin-1 (Fer-1), an inhibitor of ferroptosis is an aromatic amine that specifically binds with lipid ROS and protects cells against lipid peroxidation [49], Fer-1 was injected daily to allow viable tumors to develop. Following the establishment of tumors, Fer-1 injections were discontinued. As expected, the withdrawal of Fer-1 led to a notable decrease in the growth of the GPX4^KO^ FSP1^KO^ tumors compared to the GPX4^KO^ tumors (which continued to increase in size with the continuous use of Fer-1) [48]. We can conclude that FSP1 continue to promote the growth of H460 lung cancer tumors in vivo in the absence of GPX4. Consequently, there is no doubt that FSP1 regulates ferroptosis in lung cancer cells negatively.

### 3.7. P53

As the most extensive tumor suppressor, p53 plays a multifunctional role in controlling DNA repair, apoptosis, and cell cycle checkpoint [50]. Strong evidences hint that p53 also inhibits tumors by promoting ferroptosis. For example, p53 posttranscriptionally inhibits the expression of SLC7A11, a key component of the system x_c_^−^, which suppresses cysteine uptake and sensitizes cells to ferroptosis [51]. According to Huang et al. [52], the upregulation and activation of p53 by erastin-induced ROS contribute to ferroptosis in A549 lung cancer cells. The authors, at first, proved that ROS upregulates and activates p53 in response to erastin exposure, and that it was erastin-induced ROS that stimulated p53 in A549 cells. To explore the impact of activated p53 on erastin-induced ROS generation, the authors assessed ROS levels in A549 cells with or without p53 knockdown after erastin stimulation. Accordingly, the expression of p53 increased erastin-induced ROS generation. Additionally, erastin treatment promoted ferroptosis in A549 cells, an action significantly reversed after p53 knockdown. Eerastin exposure, therefore, led to ferroptosis that was partially dependent on p53.

## 4. Ferroptosis in Lung Cancer Therapy

### 4.1. Chemotherapy

It is clear that cisplatin-based chemotherapy (CDDP) is becoming the standard of adjuvant therapy for patients with advanced NSCLC, but this kind of chemotherapy regimens cannot effectively improve overall survival (OS) due to its apparent resistance to cisplatin [53]. Because of the shortcoming of CDDP, other avenues, including ferroptosis, have been explored to enhance treatment. Ferroptosis plays an important role in lung cancer chemotherapy; it has been shown to enhance the anticancer effect of cisplatin in the treatment of lung cancer. Guo et al. [54] observed that ferroptosis possibly become a promising approach in cancer therapies making up for the weakness of some classic drugs and openning up a new strategy for its clinical application. Specifically, the authors demonstrated that cisplatin prompted both ferroptosis and apoptosis in A549 and HCT116 cells, in which the consumption of GSH and the inactivation of GPxs play a key role in its potential mechanism. Moreover, combining cisplatin with erastin improved the antitumor activity of both substances significantly. Similarly, Zhang et al. [55] established that the inhibition of GPX4 led to ferroptosis and strengthened the anticancer effect of cisplatin in H1299, H460, and A549 lung cancer cell lines in vitro. Like in the in vitro assays, combining cisplatin with RSL3 in vivo led to a reduction in the growth of tumors more effectively than the application of cisplatin or RSL3 separately in female nude mice xenograft models. Also shown in the exploration of the potential mechanisms used by cisplatin to synergize with RSL3 was cisplatin's induction of ferroptosis via ferritinophagy. A recent investigation has even suggested that ferroptosis could play a role in cisplatin-resistant NSCLC. Per Li et al. [56] discovered that erastin/sorafenib resulted in cisplatin-resistant NSCLC cell ferroptosis via inhibiting the Nrf2/xCT pathway. Specifically, CDDP prompted the activation of the Nrf2/xCT pathway in NCI-H1299 and A549 lung cancer cell lines, with the activation level of this pathway correlates with the degree of CDDP resistance. What is more, the Nrf2/xCT expression levels in CDDP-resistant NSCLC cells increased dramatically; however, erastin and sorafenib triggered ferroptosis in these cells. Low-dose CDDP in combination with erastin/sorafenib could effectively eliminated CDDP-resistant NSCLC cells. The authors also demonstrated that erastin/sorafenib limited in vivo tumor growth in nude mice xenograft models [56], suggesting that erastin/sorafenib-induced ferroptosis may offer a new prospect for the treatment of patients diagnosed with NSCLC who have failed CDDP treatment and improve their OS..

### 4.2. Radiotherapy

Radiotherapy is one of the main strategies for lung cancer treatment [57]. However, resistance to radiation often leads to poor anticancer outcomes [58, 59]. According to Pan et al. [60], erastin resulted in reduced radioresistance in NSCLC cells partially through inducing GPX4-mediated ferroptosis. The authors used radioresistant subtypes of NSCLC cells in vitro, such as A549-R and H460-R, which were the result of high-dose hypofractionated irradiation. Cell vitality was evaluated after the treatment with erastin or irradiation (IR) separately or erastin combined with IR. Apparently, combining erastin and IR killed more cells than the application of each of the substances separately. Furthermore, erastin inhibited the GPX4 expression in NSCLC radioresistant cells, subsequently radiosensitizing the cells to radiation. Another study showed that erastin-induced and GPX4 inhibition-induced cell death was partially mitigated by the use of deferoxamine, but not Z-VAD-FMK, a caspase inhibitor [61], and olaparib, a PARP inhibitor [62], indicating that erastin and GPX4 inhibition could induce ferroptosis in radioresistant cells to exert the antitumor effect. Yuki et al. [63] also showed that erastin sensitizes cancer cells to X-ray irradiation through glutathione starvation in lung adenocarcinoma cells (NCI-H1975). The authors obtained the same results in vivo after subcutaneously inoculated NCI-H1975 cells into the left forelimb of mice under anesthesia. Erastin treatment enhanced radiotherapy and reduced glutathione concentration in a tumor xenograft model. In their most recent research, Lei et al. [64] revealed that ionizing radiation (IR) induced ferroptosis in A549/H460/H1299/H23. Mechanistically, IR induced not only the production of reactive oxygen species (ROS) but also the expression of acyl-CoA synthetase long chain family member 4 (ACSL4), a lipid metabolism enzyme required for ferroptosis. Their research found that ACSL4 defeciency largely inhibited IR-induced ferroptosis and enhanced radioresistance of cancer cells. Additionally, they further found that IR inhibited the expression of SLC7A11 and GPX4, both negatively regulating ferroptosis. The radioresistance of cancer cells was significantly reduced, and the sensitivity of xenograft tumors to IR was largely increased after inactivation of SLC7A11 or GPX4. Encouragingly, ferroptosis is also present in cancer patients undergoing radiation therapy, with higher levels of ferroptosis associated with better radiation response and longer survival.

### 4.3. Immunotherapy

There are two important biological functions of T cell death, one is the elimination of targeted cells by T cell, and the other is the elimination of T cells themselves. T cells, as major participants in adaptive immune responses and functions, are prominent in maintaining homeostasis by selectively killing infected or dysfunctional cells and play a regulatory role in cancer immunotherapy [65]. Cancer immunotherapy restores and enhances the effector function of CD8^+^ T cells in the tumor microenvironment [66]. Although there is no related in vivo or in vitro research on ferroptosis in lung cancer immunotherapy, ferroptosis, arguably, could enhance the anticancer effect of immunotherapy. According to Wang et al. [67], immunotherapy-activated CD8^+^ T cells increase the production of lipid peroxides in tumor cells, promoting ferroptosis in tumor cells, which in turn improves the antitumor effect of immunotherapy. Specifically, interferon-*γ* (IFN*γ*) released by CD8^+^ T cells downregulated the expression of system x_c_^−^[68], reducing the cell uptake of cystine, which led to a subsequent reduction in glutathione, the synthetic substrate of GPX4. Lipid peroxidation in tumor cells was, therefore, promoted, eventually triggering ferroptosis. The authors also evaluated mouse ovarian tumor ID8 cells and melanoma B16 cells and found that the synergistic effect of IFN*γ*, and cystinase caused an increase in the formation of intracellular lipid peroxides and accelerated cell ferroptosis, possibly because cystinase can significantly induce oxidative stress in cells by degrading cystine and cysteine, leading to cell death [36, 69]. Furthermore, in nude mouse xenograft models of ID8 cells, combining an immune checkpoint PD-L1 blocker with cystinase enhanced T cell-mediated antitumor immune effects and induced ferroptosis in tumor cells. The ID8 cell-derived tumor growth was similarly inhibited in mice treated with either a PD-L1 blockade or cyst(e)inase separately, but was strongly inhibited in mice treated with a combination of the two substances. Additionally, the expression of the glutamate/cystine reverse transporter was inversely associated with the recognition of CD8^+^T, the expression of IFN*γ*, and the prognosis of cancer patients. Therefore, T cell-promoting ferroptosis in tumor cells is a new type of antitumor mechanism. The combination therapy targeting the tumor cell ferroptosis pathway, particularly one that uses immune checkpoint blockers as one-half of combinations, is expected to become a new type of tumor treatment. In recent years, immunotherapy, especially PD1/PD-L1 blockers that have been widely used in clinically advanced lung cancer patients, has greatly benefited cancer patients. So, whether cystinase and PD-L1 blockers, as demonstrated by Wang et al., can enhance the lethality of T cells on lung cancer cells remains to be verified further.

## 5. Future Perspective and Conclusion

Ferroptosis is a recently identified iron-dependent programmed death that differs from apoptosis, necrosis, and autophagy and has a unique occurrence mechanism. So far, the specific mechanism of ferroptosis remains unclear, but it involves many signaling pathways and intracellular biological processes. Its importance in tumor therapy in vitro or in vivo has garnered extensive attention, with more and more targeted ferroptosis therapies being studied.

**Figure 1:**
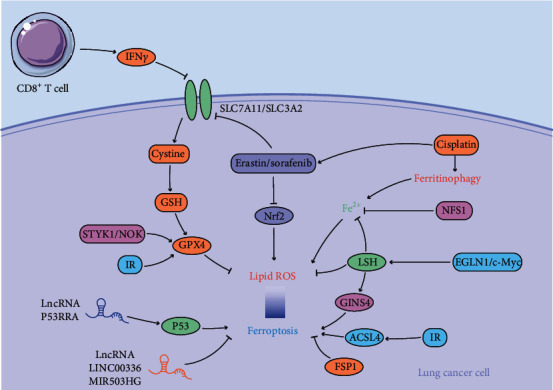
The possible mechanism of targeting ferroptosis enhancing antilung cancer. (IFN*γ*: interferon-*γ*; GSH: glutathione; LncRNA: long noncoding RNA; IR: irradiation; STYK1/NOK: serine threonine tyrosine kinase 1/novel oncogene with kinase domain; GPX4: glutathione peroxidase 4; Nrf2: nuclear factor erythroid 2-related factor 2; EGLN1/c-Myc: Egl Nine Homolog 1/c-Myc; LSH: lymphoid-specific helicase; GINS4: Go, Ichi, Nii, and San complex subunit 4; ACSL4: acyl-CoA synthetase long chain family member 4; FSP1: ferroptosis suppressor protein 1).

Lung cancer is one of the most common malignant tumors. Its incidence and mortality rates rank first among diseases in China. Since the concept of ferroptosis was first proposed by Dixon in 2012 [7], little research has been conducted on its impact on lung cancer, and the few studies that exist have focused mainly on investigations at the cellular level, such as the use of ferroptosis inducers to promote lung cancer cell death. Hence, this article focused on summarizing the hotspots and major findings in ferroptosis research in lung cancer, including the mechanism of ferroptosis in various lung cancer cell lines (Table 1) and targeting ferroptosis enhancing antilung cancer (Figure 1).

More scholars are willing to apply ferroptosis to lung cancer treatment in vitro, including targeted ferroptosis and ferroptosis combined with other anticancer methods to inhibit tumor cell growth, something quite apparent. However, more in vivo studies must be performed to validate the role of ferroptosis in the treatment of lung cancer first, and new selective ferroptosis inducers must be developed for application in chemotherapy, radiotherapy, and immunotherapy in lung cancer. As has been shown here, targeting the ferroptosis of tumor cells could become a new anticancer therapy approach in the future.

## Figures and Tables

**Table 1:** The mechanism of ferroptosis in various lung cancer cell lines.

Author year	Lung cancer cell line	Mechanism	Refs.
Bersuker et al., 2019	H460/H1703/H446	FSP1 mediates ferroptosis resistance in lung cancer	[48]
Lai et al., 2019	SW900	STYK1/NOK correlates with ferroptosis in non-small-cell lung carcinoma	[47]
Wang et al., 2019	A549/H358/PC9/SPC-A-1	Long noncoding RNA LINC00336 inhibits ferroptosis in lung cancer by functioning as a competing endogenous RNA	[39]
Gai et al., 2019	A549/H1299	Acetaminophen sensitizing erastin-induced ferroptosis via modulation of Nrf2/heme oxygenase-1 signaling pathway in non-small-cell lung cancer	[18]
Gloria et al., 2019	A549	Lysosomal destabilizing drug Siramesine and the dual tyrosine kinase inhibitor Lapatinib induce a synergistic ferroptosis through reduced heme oxygenase-1 (HO-1) levels	[44]
Yang et al., 2019	A549/H358/H522/PC9/95C/95D	LSH interacts with and stabilizes GINS4 transcript that promotes tumourigenesis in non-small-cell lung cancer	[29]
Yu et al., 2019	NCI-H1299	RNA sequencing uncovers the key long noncoding RNAs and potential molecular mechanism contributing to XAV939-mediated inhibition of non-small-cell lung cancer	[38]
Huang et al., 2018	A549	Upregulation and activation of p53 by erastin-induced reactive oxygen species contribute to cytotoxic and cytostatic effects in A549 lung cancer cells	[52]
Mao et al., 2018	A549/H358/H522/SPCA-1/PC9/95C/95D	A G3BP1-interacting lncRNA promotes ferroptosis and apoptosis in cancer via nuclear sequestration of p53	[37]
Samantha W. et al., 2017	NCI-H322/NCI-H647/NCI-H2170	NFS1 undergoes positive selection in lung tumors and protects cells from ferroptosis	[36]
Jiang et al., 2017	A549/H358/H522/PC9/95C/95D	EGLN1/c-Myc induced lymphoid-specific helicase inhibits ferroptosis through lipid metabolic gene expression changes	[28]
Pan et al., 2019	A549/H460	Erastin decreases radioresistance of NSCLC cells partially by inducing GPX4-mediated ferroptosis.	[60]
